# Improving the Adherence to Antiretroviral Therapy, a Difficult but Essential Task for a Successful HIV Treatment—Clinical Points of View and Practical Considerations

**DOI:** 10.3389/fphar.2017.00831

**Published:** 2017-11-23

**Authors:** Simona A. Iacob, Diana G. Iacob, Gheorghita Jugulete

**Affiliations:** ^1^Infectious Diseases Department, University of Medicine and Pharmacy Carol Davila, Bucharest, Romania; ^2^The National Institute of Infectious Diseases “Matei Bals”, Bucharest, Romania

**Keywords:** adherence, HIV, antiretroviral drugs, key affected population, antiretroviral treatment, adverse reactions

## Abstract

HIV infection is responsible for one the most devastating human pandemics. The advent of antiretroviral therapy has changed the course of the pandemic and saved millions of lives. Complex therapeutic regimens have been introduced since 1996 and have contributed to the transformation of HIV infection into a treatable chronic diseases. New types of potent antiretrovirals and their combinations, including “once daily” treatment, have simplified the regimens and diminished side effects. Nevertheless the adherence to antiretroviral therapy remains unsatisfactory and varies between 27 and 80% across different population in various studies, compared with the required level of 95%. The lack of adherence to antiretroviral therapy is a multi-factorial and dynamic process which raises considerable difficulties for long-term follow-up. Current solutions to this problem are complex. These should be applied by a multidisciplinary team and should take into account key features related to both the individual and social factors as well as to the population to whom it belongs (children, teenagers, elderly, marginalized population like drug users, incarcerated patients, sex workers, etc.). Importantly, adherence should continue to be monitored even in patients known to be compliant. In case of subsequent failure the team should identify the reasons for non-adherence and apply the appropriate methods. Where usual methods have no chance of success, a coordinated package of services also known as “harm reduction” can be offered in order to reduce the risks of transmission. The current article analyses the concept of adherence to antiretroviral therapy, the shortcomings of this medication and the methods that can be applied in practice to increase adherence. Emphasis is placed on the analysis of groups at high risk for HIV infection that currently represent the spearhead with which the HIV pandemic is spreading.

## Introduction

The HIV pandemics has affected over 80 million people, half of which have succumbed to the infection. Current estimates indicate that 36.7 million people living with HIV/AIDS receive antiretroviral therapy (ART). The use of highly active antiretroviral treatment (HAART) has led to a major therapeutic success, gradually altering the course of the disease and transforming HIV/AIDS into a chronically manageable disease. The complex principles of HAART have been validated and subsequently applied in the treatment of chronic viral C hepatitis as well as in other chronic diseases. This therapeutic model is based on various combinations of antivirals (ARVs) belonging to different drug classes according to predefined rules. A strict adherence to HAART strategy is required to ensure treatment success.

Currently the Joint United Nations Programme on HIV/AIDS (UNAIDS) and the World Health Organization (WHO) strive to achieve the target of 90% treatment coverage for all HIV patients as well as 90% virological success in treated patients (UNAIDS, 2015). Reaching these targets in 2020 the Programme could ultimately stop the HIV pandemics by 2030 according to UNAIDS estimates. Nevertheless, these advances should be critically viewed in terms of adherence. Achieving and maintaining virological success requires an adherence rate about 95% (Paterson et al., 2000). In view of this aspect several studies have underlined the suboptimal adherence rates toward ART in HIV patients (Bartlett, 2002) along with the major difficulties for finding efficient solutions (Chaiyachati et al., 2014).

The current chapter reviews the concept of adherence to ART, practical obstacles to a high level of adherence and the appropriate solutions.

## General issues

The first cases of AIDS were described in 1981. At that time the HIV/AIDS infection had a high rate of transmission and was universally fatal. The advent of ART had led to the paramount advance of treating HIV/AIDS as a chronic and controllable infection. Today HIV patients who benefit from correct treatment are estimated to have similar survival rates to uninfected patients (Bhaskaran et al., 2008; Wing, 2016). The long-term success of ART was noticeable after the year 2000, following the rigorous implementation of the treatment principles for various classes of ARV drugs. These precise drug combinations were outlined in 1996 as “highly active antiretroviral therapy” (HAART) and later referred to as “combined antiretroviral therapy.” However, despite the significant decrease in mortality and transmission risk, subsequent reports have shown that neither the efficacy of the drugs nor the optimized HAART principles can replace the high adherence to ART.

Thus in order to achieve undetectable viral loads HIV patients are estimated to need an adherence level above 95% (Chesney, 2003). Is such an adherence possible to current ARVs and for how long?

## Discussing the concept of adherence to ART

Adherence to ART is a pre-requisite to ensure the virologic success in HIV patients. However, the lack of consensus that has existed over the last 4 decades on the concept of adherence to medications and also different methods of analyzing this concept in clinical trials prevented a practical quantification of this concept or effective correction methods. These difficulties have ultimately led to a multitude of confusing terminologies in both the scientific papers and clinical practice. The first authorized definition proposed the term “compliance” that was associated only with the patient's ability to follow the clinical prescription. Next, the agreement between the prescriber and the patient became more and more important and the term of adherence was coined. In 1999 Horne defines adherence as “the way in which individuals judge a personal need for a medication relative to their concerns about its potential adverse effects” (Horne and Weinman, 1999). Horne's definition was later revised to “ the extent to which a person's behavior—taking medication, following a diet and/or executing lifestyle changes, corresponds with agreed recommendations from a health care provider (WHO, 2001). A new taxonomy was introduced in 2012 along with the publishing of the “Consensus on European Taxonomy and Terminology of Patient Compliance.” The new terminology was the result of a 3 year-research started by the European research groups in the field of adherence to medications and finalized through the ABC project (Ascertaining Barriers for Compliance: policies for safe, effective and cost effective use of medicines in Europe) (ABC Project Team, 2018). The ABC project took into consideration the concept of adherence as well as the factors that lower the adherence to treatment and that could be addressed by various policies. According to Vrijens (the ABC work-package leader) the project identified 3 processes that need a separate analysis: “Adherence to medications,” “Management of adherence,” and “Adherence-related sciences” (Vrijens et al., 2012). At the same time the definition of adherence has been changed in” the process by which patients take their medications as prescribed.” The concept was considered to encompass 3 distinct phases: *initiation, implementation*, and *discontinuation*. These processes can be defined as follows:

1. Initiation - “the moment when the patient takes the first dose of a prescribed medication”
2. Discontinuation - “the moment at which the patient stops taking the prescribed medication”
3. Implementation - “the extent to which a patient's actual dosing corresponds to the prescribed dosing regimen, from initiation until the last dose.”

Medication persistence defined as “the length of time on regimen before discontinuation” was considered as an important aspect of adherence.

The concept of adherence to ART is of a paramount importance for HIV treatment. This also results from its inclusion in the “HIV treatment cascade and care continuum,” a framework developed beginning with 2013 which consists of five main steps: “Diagnosis,” “Linkage to care,” “Retention in care,” “Adherence to ART” and “Viral suppression” (Kay et al., 2016).

There are 4 major factors that could influence the various phases related to ART adherence:

- *the selected ARV drug*, which could lead to various side effects and various restrictions, ultimately impacting the patient's schedule and the possibility of taking other needed medicines
- *the doctor devotion* including the time dedicated to counseling, information and establishing a trusting relationship
- *the patient*- in terms of patient's understanding, will to fight HIV and to accept ART along with its advantages and disadvantages
- *the social and family background*—able to persuade the patient to continue ART (encouragement, surveillance) or, on the contrary, to reject or discriminate the patient

Bringing together these 4 key factors represent an extremely arduous process that becomes even more difficult with the passing of time. In this regard, the adherence difficulties of HIV patients to a chronic treatment are common to other chronic diseases such as diabetes, heart or psychiatric diseases.

## Quantifying the adherence in HIV patients

The methods used to quantify the ARV adherence need address the dynamic nature of this concept. In this respect all the phases previously mentioned (initiation, implementation, and discontinuation) should be thoroughly monitored on a long period of time. Using only one method or quantifying only one phase in a restrained period of time has been shown to lead to biased results. Nevertheless, due to the multiple methods of monitoring and to the evolving definition of this concept there is currently no gold standard for measuring adherence rates. Therefore the results frequently differ depending on the chosen methodology. For example, the adherence rates are higher in reports that focus on a short period of time (previous week or up to 4 weeks before starting the study (up to 90–100% adherence) whereas studies on larger time-frames offer a more pessimistic analysis (Mills et al., 2006). Usually, however, adherence is analyzed over long periods of time.

Listed below are the most common methods used to monitor the adherence toward ART in different clinical trials:

- *Self-report medication adherence* is a simple method, albeit with the disadvantage of overestimating the adherence behavior compared with other assessment methods (Wagner and Rabkin, 2000). It retains a good specificity (i.e., positive predictive value) and weak sensitivity (i.e., negative predictive value). Still, it is considered a reliable method according to a meta-analysis on 42 studies (Shi et al., 2010). The use of standardized, self-reported questionnaires (“Morisky Medication Adherence Scale, MMAS”) favors a better evaluation of the adherence rate as well as the possibility to make valid comparisons between different studies (Gokarn et al., 2012). A complete questionnaire entails the ARV regimen: dose, interval between doses, administration route, number of days with incorrect administration, respecting the prescriptions. Notably, the type and complexity of questionnaire could significantly influence the result of the study. Self-report of adherence are probably the most commonly used study method for quantifying the adherence in HIV patients. Its implementation is easy, rapid, flexible and inexpensive and it is plausible despite the limitations that could arise from the confidence in various answers (Stirratt et al., 2015). Furthermore this method has the advantage of correctly estimating the initiation and implementation phase. Nonetheless it is inadequate regarding the ARV discontinuation and the persistence with therapy toward which the patient might be voluntary or involuntary biased.
- *Pharmacy Refill Data*. Non-adherence can be objectively analyzed using pharmacy records. The analysis of this data offers reliable comparisons and has been previously used in various key studies (Steiner and Prochazka A, 1997; Wood et al., 2003; Grossberg and Gross, 2007; Haberer et al., 2017). However the use of this method does not provide accurate data on the patient's real commitment regarding treatment adherence.
- *Medication Event Monitoring System* (MEMS) is an electronic monitoring system involving a wireless pillbox that tracks the exact date and time each time it is opened and closed. The technique is objective and it is more sensitive than self-reports (Arnsten et al., 2001). Nevertheless, the method is only useful in regimens in which the patient opens the box once daily and it is inadequate when the regimen entails the administration of multiple tablets on the same dose (an aspect commonly encountered in most ART regimens). Although, it is currently mostly used as an experimental method, this type of real-time adherence monitoring could become a preferred method of analysis even in resource-limited settings according to Haberer (Haberer et al., 2010).
- *Therapeutic drug monitoring* relies on the analysis of serum concentrations for various ARVs. It is a costly method and the results need to be interpreted in accordance with the pharmacokinetics of various drugs (for example the serum concentrations of some ARV like nucleoside analogs, might not reflect the intracellular drug concentrations).

This method is only adequate for determining the serum level of recently administered ARVs and offers no indication on previously administered drugs and neither does it help in the event that various ARVs have been periodically discontinued before the current administration.

Notably, a study performed on 230 patients followed for 48 weeks suggested a better predictability for pharmacy refill data and electronic devices toward self-reports (Orrell et al., 2017). On a similar note, a recent meta-analysis on different methods and their effectiveness disclosed the superiority of electronic monitoring compared with other methods (Conn and Ruppar, 2017).

## Adherence to ART in the specific subpopulations

The level of adherence to ART differs depending on the population group. A meta-analysis on 84 observational studies in 2011 disclosed that roughly only half (62%) of HIV positive patients achieved an adherence rate of 90% (Ortego et al., 2011). There is still a high disproportion between various groups that need to be analyzed separately.

### The group of children

The adherence to ART is higher in children. Several studies from 2012 to 2014 documented an adherence rate as high as 95% in 80.9% and respectively 78.6% of the HIV infected children (Azmeraw and Wasie, 2012; Arage et al., 2014). Still, children remain a vulnerable group, depending on the permanent adult care and family support as well as the availability of convenient and efficient ARV formulations.

### The group of teenagers

A study in teenagers by Kim et al in 2014 (Kim et al., 2014) indicates a large variability, ranging from an adherence rate above 70–85% in young people from Africa/Asia to only 50–60% in those from Europe and North America. In this group ART discontinuation is frequent. The relevance of the teenagers group is extremely important for the subsequent evolution of HIV, as this group represents over 40% of the new HIV infections and the most active population in what concerns sexual transmission. Lack of ARV adherence of this group is worrying and is based on factors that act simultaneously at this age: the fear of disclosure and social stigma, low social support, inadequate communication and education, the entry in various delinquent or inappropriate social groups, the lack of a motivation and the depression related to living with HIV/AIDS.

### The group of women

All women including the pregnant women are at a higher risk of non-adherence compared with their counterparts (Ortego et al., 2012). In this group, the initiation of ART and the implementation phase may be deficient either because of fears of being detected with HIV and discriminated either due to lack of time needed for medical care. Studies on pregnant women also show high discrepancies depending on the location (urban/rural) and healthcare settings. In rural and low-income settings, only 1 in 122 mother-child pairs met the 95% adherence threshold (Kirsten et al., 2011); in studies with a satisfactory counseling and monitoring, the adherence levels have reached 87.1% (Ebuy et al., 2015).

### The group of elderly people

Elderly (over the age of 50 years) represents an increasing population in the following years. In some areas this group has already reached 50% of the local HIV population (UNAIDS, 2013; Wing, 2016) and is predicted to rise in the following years. In a study by Johnson et al 70–80% of the analyzed population displayed an adherence rate above 90% (Johnson et al., 2009) while other reports indicated overall adherence rates up to 87% (Hinkin et al., 2004). The use of recreational drugs and alcohol has been shown to lower the adherence below 70% (Gonzalez et al., 2011). The screening for these risk factors should not be neglected in this population. Furthermore, poly-pharmacy and depression frequently found in older patients have a negative effect on ARV implementation lowering the adherence throughout time. Therefore, individual differences should not be neglected despite the preliminary favorable data on adherence in this group.

### Key populations

Female sex workers represent a vulnerable group with a high and worrisome variability in what concerns the adherence to treatment. In a meta-analysis from 2014, Mountain et al. (2014) discusses the discrepancy rates between high-income countries where adherence rates reach 80% and low and middle-income countries where the adherence is as low as 36%. Female sex workers are a key population regarding HIV transmission and therefore can benefit from an adequate surveillance in what concerns counseling, support groups and/or directly administered therapy. In the settings where such a surveillance is ensured, this group display a higher adherence compared with other women (Ortego et al., 2012). Nevertheless this group encounters numerous obstacles to start ART (fear of HIV stigma and being known as a sex worker or the marginalization by health care providers) as well as to respect ART's recommendations and afford the financial costs for medical care (Mountain et al., 2014).

On the other hand other key groups such as men who have sex with men (MSM) as well as lesbian, gay, bisexual, transsexual, intersex (LGBTIQ) communities exhibit a global HIV prevalence up to 19 times higher than previous groups (UNAIDS, 2014). These groups are particularly difficult to analyse. There are few studies and the financial resources dedicated to this community are scarce. In a study from 2014, only 45% of MSM reported a good adherence to ART (Liu et al., 2014). Various factors are responsible for these low rates such as HIV stigma, social isolation, difficulties to access health-care programmes, fear of health-care seeking as well as denial of care, depression and the lack of data regarding drug interactions between hormonal treatment and ART (Graham et al., 2013).

### Positive drug users

It is estimated that 1 in every 10 new HIV infected patients globally is an injecting drug user (IDU). This key risk-group accounts for the increasing number of new HIV cases especially in Central Asia and Eastern Europe where it designates roughly half of the new cases. Due to drug dependency issues and due to the large diversity of illicit drugs, the management of this population is particularly challenging. The adherence is low even in optimistic studies, citing a 63% adherence at most (Hinkin et al., 2007). The choice of the recreational drug appears to play a significant role on the adherence to ART. In a study on cocaine abuse, the IDUs consuming cocaine exhibited much lower adherence rates (27%) compared with non-cocaine users with a 68% adherence rate (Arnsten et al., 2002). This observation is particularly worrisome in view of the extremely wide use of crack coca (Wechsberg et al., 2012) and of the sexual risk behaviors of IDUs for HIV transmission (Inciardi, 1995). IDUs subscribe to multiple non-adherent behaviors—from forgetting one pill to abandoning treatment for variable periods of time. Adherence is strongly influenced by multiple psychological factors (depression, cognitive impairment especially in patients with a strong methamphetamine use, psychiatric disturbances and lack of social support). The accurate follow-up and monitoring of these patients is particularly difficult and many IDUs abandon treatment completely as the hectic lifestyle of these patients is in strong disagreement with the strict discipline required by HAART.

### Incarcerated people

Incarcerated people add to the populations with a low adherence rate (54%) and with few interventional possibilities to reverse their deviant behavior (Uthman et al., 2017). Directly observed therapy applied in correctional institutions have so far shown an inadequate increase of adherence rates (Wohl et al., 2003).

### Socially isolated people

Socially isolated people have a high risk of non-adherence and ART failure (Reblin and Uchino, 2008). Social support (social networks, relationships-friends and relatives) as well as the emotional support provided by HIV support groups are essential pillars in the care of HIV patients (McCoy et al., 2009). Unfortunately, enacted HIV stigma frequently leads to an alienation of the patient from close friends as well as to the withdrawal of the social support for the stigmatized patient (Takada et al., 2014). Subsequently social stigma leads to various serious issues on adherence and treatment monitoring, including a significantly higher risk of death (Elovainio et al., 2017).

Although each of the aforementioned populations exhibit specific traits, the adherence to ART is a common issue risking to invalidate the benefits of this therapy. Therefore it is essential to understand the particular features of ART accounting for these low adherence rates.

## Barriers to ART

### The complexity of art regimens

The most relevant disadvantage of ART regimens is their complexity. ART principles require the combination of more ARVs, usually from three different ARV classes targeting at least two phases of viral replication. Currently the U.S. Food and Drug Administration has approved 26 ARV agents from six different ARV classes, as well as three “fixed-dose combination” with one-daily administration (“single tablet regimen”), namely “efavirenz/emtricitabine/tenofovir,” “elvitegravir/cobicistat/emtricitabine/tenofovir disoproxilfumarate” and “elvitegravir/cobicistat/emtricitabine/tenofovir alafenamide.”

Although the efficacy of ART is indisputable, no antiviral regimen can cure the disease. This aspect is explained by the inevitable resistance that occurs during ART, the HIV integration in the cellular genome and the presence of HIV “sanctuaries” that impede the complete elimination of HIV. Therefore, the goal of ART is to rebuild the immune response, and maintain the plasma viral load to an undetectable level (< 50 copies/mL), thus preventing the progression and transmission of HIV infection. In the absence of correct treatment and adequate prophylactic interventions, infected patients can further transmit the disease.

Most of ART regimens of first intention combine two representatives from one ARV class with one of a different class. In this circumstance the total number of recommended tables per day varies from one to six. However, “fixed-dose combination” or “single tablet regimens” are available in few countries only, while low income countries with the highest number of cases continue to rely on regimens that entail a large pill-burden.

ART regimens can be divided between “preferred regimens” considered as “first line regimens” that have high efficacy and low toxicity and “alternative regimens” that are usually cheaper but with higher toxicity and higher pill-burden. These classification take into account objective criteria such as the HIV/AIDS status and treatment costs. The ARV regimen once established needs to be strictly monitored in terms of effectiveness and relevant side-effects. It needs to be changed in the case of drug toxicity and ARV resistance. Furthermore various changes can be made depending on the patient (age, social category, life style, the ability to understand recommendations etc.). Therefore, ARV regimens need to be continuously changed and adapted to various external factors.

Still, the large number of pills is probably the most common complaint of HIV-patients. A meta-analysis performed on studies between 2005 and 2014 shows a significantly higher adherence in patients with a once-daily fixed-dose (“single tablet regimen”) compared to any other treatment regimen (Clay et al., 2015). Once-daily fixed dose regimens have been proved to be a crucial step to simplify ARV treatment and are currently the easiest method to increase adherence. Another prospective alternative in this direction is given by the promising long-lasting ARV agents that enable an injectable treatment every 1–3 months. These ARV drugs are under evaluation and appear to have no significant side effects (Llibre et al., 2017).

Other barriers to ART entail the complex drug regimens needed for multiple comorbidities associated with HIV infection. These patients often need more medications than non-HIV infected patients (Gimeno-Gracia et al., 2015) and their associated treatments become more and more complex with the passing of time. Thus, even patients willing to keep a strict schedule and not to forget the doses could involuntarily miss doses, modify or even abandon various drugs. Sometimes this risk could exist from the beginning, in other cases this could arise with the passing of time and with ensuing co-medications. Multiple drugs co-administered with ART are an important predictor of non-adherence due to the associated pill-burden, side effects and drug interactions (Cantudo-Cuenca et al., 2014).

### Side effects of art

No drugs lack side effects. Adverse effects of ARVs are related to each compound as well as to the genetic factors of the host and the patient's lifestyle. In a study by Golrokhy et al (Golrokhy et al., 2017) 94% of ART exposed patients exhibited adverse effects. The discomfort created by various side effects is an important factor that diminishes the adherence or leads to treatment discontinuation.

In order to understand the difficulty of ART regimens in HIV infected patients we need to understand the particular features of ARV drugs. Listed below are some of the most important side effects of the main ARV drugs that could have a deleterious effect on the persistence phase of adherence (Margolis et al., 2014).

### The lipodystrophy

This late complication results in morphologic changes in the fat distribution with central obesity and localized lipoatrophy (17–83% of patients). This physical transformation leads to depression and frustration, contributes to a poor self-image and ultimately results in the patient's abandonment of treatment and loss from care (Carr et al., 2000). Lipodistrophy occurs after at least 2 years of ARV administration and is common in patients using nucleoside reverse transcriptase inhibitors (NRTIs) or protease inhibitors (PIs), two of the most recommended ARV classes. The risk is higher in patients using ARVs from both classes. It is noteworthy that these combinations are found in multiple first-line regimens.

### Systemic side effects

These include: adverse cardiovascular events including the risk of myocardial infarction, peripheral neuropathy, pancreatitis, bone marrow suppression, myopathy, renal toxicity, osteoporosis, and hypersensitivity reactions. These are most frequent in NRTIs regimens but could also appear due to other drug classes. NRTIs are a common denominator of almost all ART regimens. Except for hypersensitivity reactions, most side effects appear after prolonged periods of time and the patient might not realize the connection with the ARV treatment.

### Neurological effects

Patients complain of nightmares, insomnia and up to 5% decide to discontinue the medication. These complains are characteristically of efavirenz, a first-line ARV belonging to non-nucleoside reverse transcriptase inhibitors (NNRTIs). Neuropsychiatric side effects to efavirenz were noted repeatedly as causes of non-adherence.

### Gastrointestinal intolerance

Although these characteristic side effects of protease inhibitors class (PIs). are usually mild, their persistence could determine the refusal and disregard of treatment by children and pregnant women.

There are various ARV drugs that target unpleasant effects, persist and worsen on the long-term due to the long-term use of ART. Some effects such as allergic and digestive side effects appear immediately and can be rapidly addressed. If solved promptly these issues do not alter the patient's adherence to treatment. Other side effects, such as lypodystrophy, appear after prolonged use of ART and result in upsetting physical transformations.

All these situations require a trusting, efficient and permanent dialog with the health care provider.

### Side effects that arise due to drug or food interactions

One example is that of the use of PIs. Efficient serum levels of PIs can be reached only in the presence of certain meals (such as those with a high level of fat). Ignoring these administration constrains leads to therapeutic failure and represents a form of non-adherence that is hard to be recognized. Furthermore the serum level of PIs can be reduced by a concomitant administration of various compounds such as garlic, hypoxis hemerocallidea and hypericum perforatum (medicinal herbs), sutherlandia (a traditional herb used for depression) and some vitamins (thiamine, riboflavin). At the same time, limited access to food is another objective reason for disrespecting the indications of ARVs administration in low-income countries. Otherwise, most dietary recommendations are disrespected and represent a type of non-adherence.

Alcohol abuse also impacts adherence either directly (for example through neurological impairment) or indirectly by ARV interaction with alcohol consumption and the patient's lack of willingness to stop the alcohol (Cook et al., 2001).

With time HIV patients could feel trapped by a treatment that gives them a sense of being vulnerable. They ultimately isolate themselves, lose trust and abandon treatment.

Medical charts for tracking side effects are an easy method to identify the multiple side-effects to ARV treatment (Menezes de Pádua and Moura, 2014). One can note that side effects are various and metabolic disturbances can be hard to accept by some patients. Even moderate side effects pose important difficulties to a high adherence. Fortunately these effects can be commonly prevented or diminished with specific treatment. At the same time, if the patient is not well instructed he could independently decide to stop the disturbing drug either periodically or indefinitely. Therefore an efficient follow-up and a permanent counseling of these patients is essential for maintaining the treatment regimens despite side-effects, food restrictions, drug interactions and drug-fatigue. The psychological support is particularly difficult in front of these difficulties.

## Analysing the lack of adherence to ART

A low adherence to treatment is common in chronic diseases where patients depend on multiple treatment regimens. The adherence to ART is not related to one single factor, but it is rather multifactorial. Some of these issues only act at certain times and their intensity and importance can vary throughout time. Moreover, the same individual can exhibit a differential behavior to certain external factors.

The lack of adherence can be voluntarily (“intentional non-adherence”), in which case the patient decides not to follow treatment or involuntarily (“unintentional non-adherence”) in which case the patient does not understand or cannot respect all the indications.

### Intentional non-adherence

Intentional non-adherence arises from a variety of causes such as denial of diagnosis, lack of trust in the health-care provider and in the treatment itself, fear of HIV stigma, restraints due to a life-long treatment, difficulties to integrate the treatment into the daily routine and disappointment due to the impossibility of cure HIV infection.

### Unintentional non-adherence

Unintentional non-adherence frequently arises from misunderstanding or ignoring treatment indications. Such examples include the forgetting of doses or the voluntary change of frequency of administration. Predictors of unintentional non-adherence include the cognitive limitations, polypharmacy, the patient's personality as well as comorbidities and associated treatments. Drug and alcohol abuse are important factors leading to a decrease adherence (Millar et al., 2017). On the other hand it is debatable how and if various neurocognitive disorders impact the adherence (Kelly et al., 2014).

Overall, the most frequent reasons given for the lack of adherence include “forgetting the doses” in 35–52% of patients (Kalichman et al., 1999; Gifford et al., 2000; Nieuwkerk et al., 2001), “being away from home” in 46% of patients (Gifford et al., 2000) and “a change in the daily routine” in 45% of patients (Gifford et al., 2000). Despite its importance, the depression is much less cited and recognized as a cause of non-adherence by both the patient and the provider 9% (Kalichman et al., 1999).

The analysis of a small group with a 100% ART adherence highlighted the optimism of these patients toward ART, a very efficient doctor-patient relationship, the lack of ARV side-effects and ART confidence as a result of improved clinical, immunological and virological parameters (Sidat et al., 2007).

Nevertheless, even patients with a high degree of adherence may gradually become more inattentive to the treatment regimen considered responsible for various restrictions and a dramatic decline of their life style.

## Interventions to increase the adherence to ART

Adherence is a complex and dynamic process. As in other chronic diseases, patients with HIV experience difficulties to achieve and maintain a high adherence indefinitely. It is for this reason that the health-care provider should acknowledge that no patient can display a perfect adherence. A permanent monitoring of the patient's adherence is required at all times. Medical staff should not rush discussions on adherence even in patient's known to be compliant as it is never too late for the patient to become non-adherent.

The main issues that need to be established when monitoring drug adherence include:

- rapid identification of non-adherent patients
- establishing the causes
- finding the adequate solutions

Addressing these points requires the development of a dedicated multidisciplinary team involving the doctor and the patient, the pharmacist, the psychologist and other close relatives or friends.

Below are described the most notable interventions (common and particular to certain groups) that could improve adherence in HIV-infected patients.

## Common approaches

### Intentional non-adherence

Intentional non-adherence can be prevented through a good communication with the patient, motivational interviewing techniques and psychological therapy. The patient should be aware of the gravity of the disease and of the consequences arising from non-adherence.

*Unintentional Non-Adherence*. Unintentional non-adherence is hard to encounter and needs a complex intervention. The common approaches to maintaining good adherence are listed below:

- *Essentially is the Unambiguous and Thorough Education of the Patient about the Role of Art*. In these circumstances the psychologist needs to cooperate with the physician in order to stress the role of ART for an adequate immunologic and virological control. Commonly used methods include educational resources either orally or in a written form according to the patient's level of knowledge. The patients willing to find adequate information on adherence can also use online resources such as http://www.aidsinfonet.org/fact_sheets/view/40561).
- *The Simplification of Current Regimens*. This is a prerequisite especially in patients with multiple comorbidities and poly-pharmacy. In order to overcome the difficulties related to the pill-burden of HAART, HIV specialists have introduced the concept of monotherapy or dual maintenance regimens - drug combinations that entail single or dual drug combinations for some patients (Bierman et al., 2009). This concept was experimentally studied in patients with long term viral suppression and it is believed that could be applied in certain subpopulations in order to reduce the costs and enhance the adherence to ART. Nevertheless these therapeutic alternatives exhibit a high risk of emerging resistant mutants, so that the use of mono- or dual therapy in HIV patients to maximize adherence remains debatable.
- *Establishing a Convenient Strategy Regarding the ARV Regiment Together With the Patient*. The chosen drug regimen should be clear, simple and well organized (pill organized could also be used to ease its implementation) and easy to integrate in the patient's daily routine. Further engaging the patient to devise and to individualize the treatment could help increase the adherence.
- *Technological reminders could be employed to help* the patient to understand the importance of a rigorous schedule for the administration of ART. These tracking devised could be simple (calendars, medication checklists) or more complex (phone call reminders, SMS reminding- audio-visual or electronic texts).
- *Social protection* is mandatory for certain groups (“cash and care” protection, food security (2 meals/day), HIV support groups, parental supervision for adolescents etc.) (Cluver et al., 2016).
- *Employing the supportive care from the family, friends, pharmacists, nurses, and physicians*. The involvement of family and friends is of crucial importance to help the patient deal with stigma, social isolation and loneliness, lack of motivation and pill-fatigue.

It is hard to establish which are the most efficiently methods on adherence. There are numerous studies on increasing the adherence but comparing the results is problematic and each of these interventions should be tailored to the patient. There are no universal solutions for solving adherence. The more methods one uses the more efficient the result.

In addition to the classical methods for monitoring adherence, certain risk-groups benefit from specific techniques to increase adherence.

## Specific methods to increase adherence to ART

### Directly observed therapy (DOT)

DOT is the application of the anti-tuberculous model of directly supervised treatment to HIV patients. Current data on the efficiency of this method is contradictory, ranging from positive results (Altice et al., 2007; Macalino et al., 2007) to negative outcomes (Ford et al., 2009). In the case of various marginalized groups such as drug users or homeless people, this method could prove effective and has been shown to increase the relative rate of viral suppression from 31 to 71% (Bangsberg et al., 2001; Flanigan and Mitty, 2006). The integration of DOT in health care services for the treatment of both HIV and drug abuse could prove cost-effective. Another potential use of DOT is that of implementing this method along with long-acting injectable ARVs in these risk-groups.

### Opioid substitution treatment

Drug abuse is a major predictor of non-adherence in HIV patients (Wood et al., 2003). Most of these patients abandon treatment and are rapidly lost to follow-up. Improving the access and adherence to ARVs for drug abusers represents a difficult task for any medical service and special interventions are needed to care for these risk-group. Multiple studies have underlined that the treatment of substance abuse is a crucial step for the success of HAART (Hinkin et al., 2007). Starting treatment with methadone is probably the only high impact measure to increase adherence in this group (Hinkin et al., 2007; Spire et al., 2007; Hayashi et al., 2016). Additional factors involve family surveillance, psychiatric therapy (especially concerning depression) and concomitant alcohol withdrawal. Long-acting ARVs could significantly improve the adherence, especially when associated with DOT. This patient category could also benefit from other preventive measures such as those related to sexual transmission (motivational interviewing, protected sex) or interactive methods to support ART adherence (automated interactive voice response systems) (Aharonovich et al., 2012).

### Harm reduction

This pragmatic concept is less known in middle or low-income settings. Nevertheless this could prove one the most important actions to lower the risk of HIV infection in injection drug users (IDUs). Harm reduction refers to a coordinated package of services (education, needle syringe programmes, drug treatment, social support) offered to drug abusers. Since the reduction of drug abuse is a difficult objective, it is considered that such actions could actively reduce the risks of transmission of HIV and other viral co-infections. When applied, these programmes improved the adherence to treatment and these positive results were confirmed throughout long periods of time, exceeding even 10 years in some studies (Lambers et al., 2011; Fernandes et al., 2017). Despite being accepted in numerous countries and being recognized by WHO and UNICEF, the concept of harm reduction is still hard to implement.

### The treatment of depression

At least half of HIV patients suffer from depression according to a large study on 1,713 patients (Yun et al., 2005). Depression is a strong risk-factor for non-adherence according to numerous studies (Langebeek et al., 2014). The use of anti-depressives could significantly improve the adherence to ART even in complex ARV regimens (Kumar and Encinosa, 2009). A close collaboration with a psychiatric service and psychotherapy sessions are therefore needed in all HIV patients irrespective of age or risk group.

### The treatment of alcohol addiction

Almost half of the HIV patients admit to consume alcohol frequently and 8% are heavy drinkers (Galvan et al., 2002). The risk of non-adherence in such settings has been estimated to rise by 4.7 times. Notably, even small quantities of alcohol can lead to non-adherence (Samet et al., 2004; Barai et al., 2017). Nevertheless, behavioral interventions have not proved effective and pharmacological interventions are rarely used to decrease alcohol addiction.

### Ensuring the social support

This factor cannot be stressed enough. Social support is essential for an adequate adherence. It requires the involvement of the community through informational and emotional support as well as instrumental support (financial assistance and help with various tasks) (Berkman and Syme, 1979). Prospective developments in this direction include a specialized assistance, targeted policies and financial support. We can state that this type of intervention is presently neglected almost entirely in poorer countries and is fairly limited in other countries.

## Managing the adherence to ART in HIV infected patients

Management of adherence should be implemented through specific intervention according to the three phases recognized by the new taxonomy for defining adherence and also the concept of “HIV care continuum” (Vrijens et al., 2012; Kay et al., 2016).

### Interventions at the initiation of ARV treatment

A particular aspect of HIV patients is to defer ART over longer periods of time on a voluntary basis. The delay could be explained by various perceived barriers, such as fear of HIV, risk of stigma, lack of trust in the doctor or even denial of diagnosis and in some countries poor access to health services. Failure to ensure the “linkage to care” could take up to 3 months according to the “HIV care continuum” model (Centers for Disease Control Prevention, 2014). Interventions that could increase the initiation of ART at this stage are related to the timely identification of these patients and the initiation of ART through interdisciplinary co-operation, if necessary (e.g., psychiatric consultation).

ART should be started with a convenient administration schedule. The treatment thus offered should correspond to the patient's lifestyle and to be adapted to the patient's intellectual and social level. Guideline indications if applied should also be individualized. All the time the patient need moral support and encouragement on the success of therapy. Notably, the treating physician is the key figure ensuring the adherence to treatment in this phase. The family and social support is also welcomed.

### Interventions during the implementation phase

The implementation phase (corresponding to “the retention in care” from the “HIV care continuum” model) has received the most intense analysis regarding the adherence. During this period the patient experiences various psychological and social barriers (concerns about confidentiality, lack of support from partners and/or family, depression, social stigma etc.) as well as obstacles related to the costs of the treatment (transport to the clinic or even pill cost *per se*), difficulties to abandon various habits (e.g., alcohol consumption) and to adapt to ART side effects.

Social interventions of this period need to be tailored to individual needs providing support to combat stigma and promoting a positive approach to living with HIV. Careful planning of the medical visits is also important so as to ensure a permanent feedback between the patient and the treating physician. Interdisciplinary consults and indicated treatments could also prevent or reduce various side-effects and alleviate the patient's anxiety. Social support, food security or the “cash plus care” system are particularly relevant in poor countries (Cluver et al., 2014, 2016).

Among the methods addressed directly to the patient we mention: patient education on HIV course and adherence to ART, positive thinking, technological reminders and HIV support groups organized by hospitals involved in HIV management. Vulnerable groups must be a permanent target of these interventions.

### Interventions for preventing treatment discontinuation

All interventions applied in this phase intend to encourage the patient and to offer an adequate moral, social, and family support to prevent the patient's discontinuation of ART. Furthermore, the treating physician is responsible for observing various health-care related causes of the decrease of adherence and for establishing appropriate treatment (changing the treatment regimen, treating side-effects or other comorbidities, encouraging the patient to abstain from alcohol and/or drugs). Unfortunately, some of the obstacles and particularly those related to financial constraints cannot be easily solved and could cause treatment discontinuation (Wanchu et al., 2007).

Recent debates stress the need to implement monotherapy in restricted HIV populations or to prevent HIV infection through harm reduction programmes. Suppositions that the high efficiency of modern therapy can be attained even with lower adherence rates such as 80% (Viswanathan et al., 2015) are unconfirmed and the emerging risks from generalized such concepts remain unknown.

## Conclusions

HIV/AIDS entails one of the most challenging therapies compared with other chronic diseases. The adherence of HIV patients to ART is hard to assess due to the multiple psychological and social implications characteristic to the diverse populations exposed to HIV. Each patient has an individual motivation to follow or not to follow ART. Added to this are factors related to the complexity of the therapeutic regimens, side effects, personal habits, consumption of illicit drugs, depression, other comorbidities or other complications specific for each patient. Adherence management should address to all of these aspects and must have a social, familial, personal and health care involvement. Finally, the adherence issues revolve around so many factors that it is an extremely difficult and complex assignment to achieve the required 95% adherence rate for an indefinite period of time.

However, despite the large literature on the low adherence to ART, there are still a number of positive interventional measures that can sustain the patient's hope in a better life. The success of ART is highly dependent on the prospective application of these methods.

## Author contributions

SI, DI, GJ all contributed to the conceptualization, drafting, and editing of the manuscript. All authors read, critically revised and approved the final manuscript.

### Conflict of interest statement

The authors declare that the research was conducted in the absence of any commercial or financial relationships that could be construed as a potential conflict of interest.
